# Supplementary material for: Structural variant evolution after telomere crisis
Source: Nat Commun. 2021 Apr 7;12:2093. doi: 10.1038/s41467-021-21933-7 (PMC8027843; doi:10.1038/s41467-021-21933-7)
Supplement: Supplementary file 1 — Supplementary Information [file 41467_2021_21933_MOESM1_ESM.pdf]

Structural variant evolution after telomere crisis

Supplementary Information

- Supplementary Figures
  - Supplementary Figure 1
  - Supplementary Figure 2
  - Supplementary Figure 3
  - Supplementary Figure 4
  - Supplementary Figure 5
  - Supplementary Figure 6
  - Supplementary Figure 7
- Supplementary Tables
  - Supplementary Table 1
  - Supplementary Table 2
  - Supplementary Table 3

Dewhurst, Yao *et al*. Supplementary Figure 1

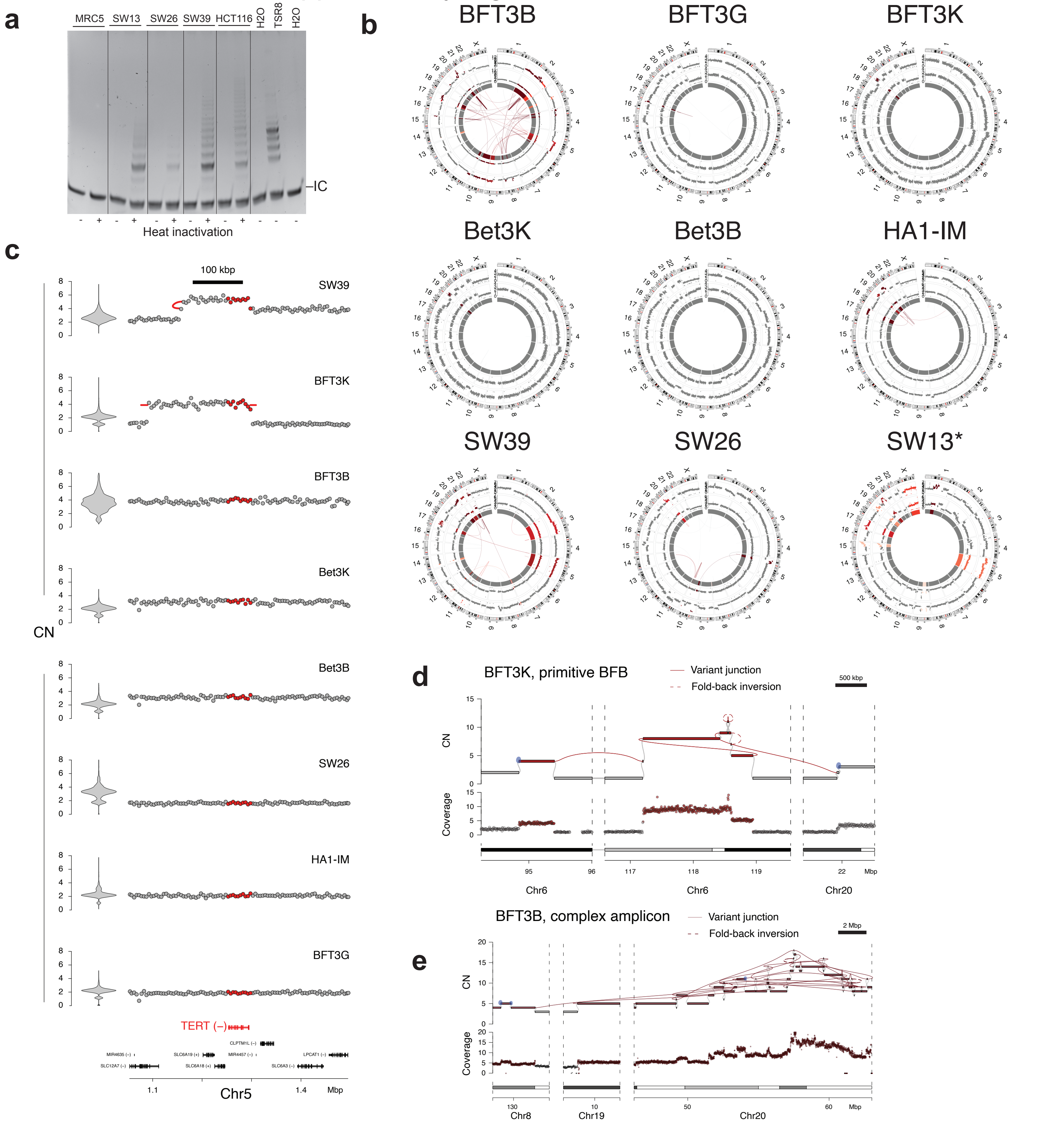

Supplementary Figure 1. Telomerase activity and complex gains after spontaneous telomere crisis resolution. Related to Figure 1.
a) TRAP assay showing telomerase activity in SV40 immortalized clones SW13, SW26 and SW39. MRC5 is included as a negative control, and HCT116 as a positive control. TSR8 is the positive control template. IC = internal control at 36 bp. Gel shown is representative, experiment was carried out at least twice. **b)** CIRCOS plots of all nine SV40 immortalized clones together with their respective pre-crisis clones. From innermost circle, variant junctions present only in post-crisis sample, colored by complex amplicon clusters or grey if not classified into any, JaBbA inferred copy number, purity-ploidy transformed binned coverage of the pre-crisis clone, of the post-crisis clone, and chromosome bands. SW13 is marked with an asterisk due to the presence of extensive pre-crisis structural rearrangements. **c)** Read depth and junction patterns at the TERT locus across the seven post-crisis clones. Each track shows binned purity- and ploidy-transformed read depth in units of CN, with variant (rearrangement) junctions and loose ends plotted as red arcs. The bottom track highlights the TERT gene among genes on chromosome 5p15. Violin plots to the left of tracks show the genome-wide distribution of read depth in units of CN, demonstrating that 6 of 7 clones have elevated CN at the TERT locus. **d-e)** Example clusters of complex gains from the cell lines shown in Figure 1A, each showing binned purity- and ploidy- transformed read-depth, with the top track showing the associated junction-balanced genome graph (see Methods3, with y-axis representing units of per cell copy number (CN) across bins and graph nodes (i.e. intervals). Grey and colored edges represent reference and variant junctions, respectively. Dashed colored edges represent fold-back inversion junctions. Blue edges represent loose ends (see Methods for further details). Bins and junctions are colored as per Figure 1A. In e, only an excerpt of the entire complex amplicon cluster proximal to chromosome 20q peak is represented.

Dewhurst, Yao *et al*. Supplementary Figure 2

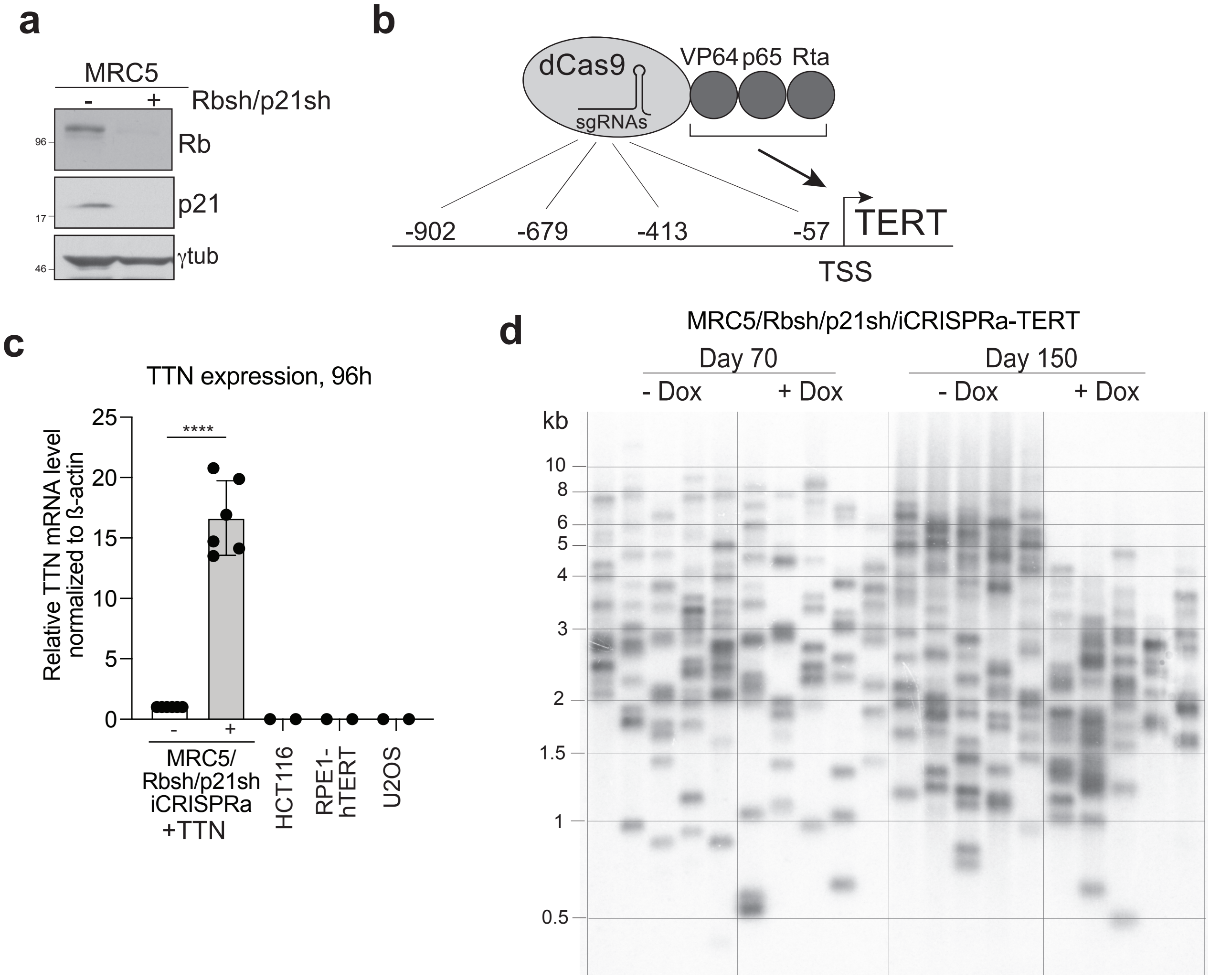

Supplementary Figure 2. A system for controlled escape from telomere crisis. Related to Figure 2.

a) Immunoblot for Rb and p21 in MRC5 cells and MRC5/Rbsh/p21sh cells. A representative blot is shown, experiment was carried out at least twice. **b)** Schematic diagram illustrating the iCRISPRa-TERT system. The positions of the TERT activating gRNAs relative to the transcriptional start site (TSS) of the TERT gene are shown. **c)** qPCR for the TTN control gene, activated with a combination of four sgRNAs in MRC5/Rbsh/p21sh/iCRISPRa cells (with or without dox, 96 hrs) n=6. HCT116, RPE1-hTERT and U2OS cells are included as negative controls (n=2). Data for dox- and dox+ samples were compared with a two-tailed student's t-test, $p<0.0001$. Error bars indicate mean ±SD. **d)** STELA of the XpYp telomere in MRC5/Rbsh/p21sh/iCRISPRa-TERT cells after 70 days or 150 days of continuous culture with or without doxycycline. This is a biological replicate of the STELA in Figure 2E.

Dewhurst, Yao *et al*. Supplementary Figure 3

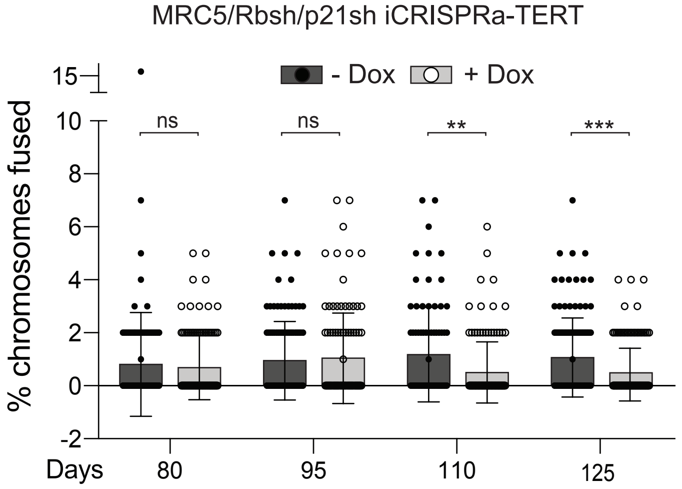

Supplementary Figure 3. Telomere fusions after controlled escape from crisis. Related to Figure 3.
Quantification of percentage of fused chromosomes after the indicated days of continuous culture for MRC5/Rbsh/p21sh/iCRISPRa-TERT cells with and without doxycycline. Error bars represent means ±SDs from three independent biological replicates, bar at mean, each dot represents one metaphase spread, n=84-127. P values derived from a two-sided student's t-test. ns, not significant; **, p=0.0012; ***; p=0.0004.

Dewhurst, Yao *et al*. Supplementary Figure 4

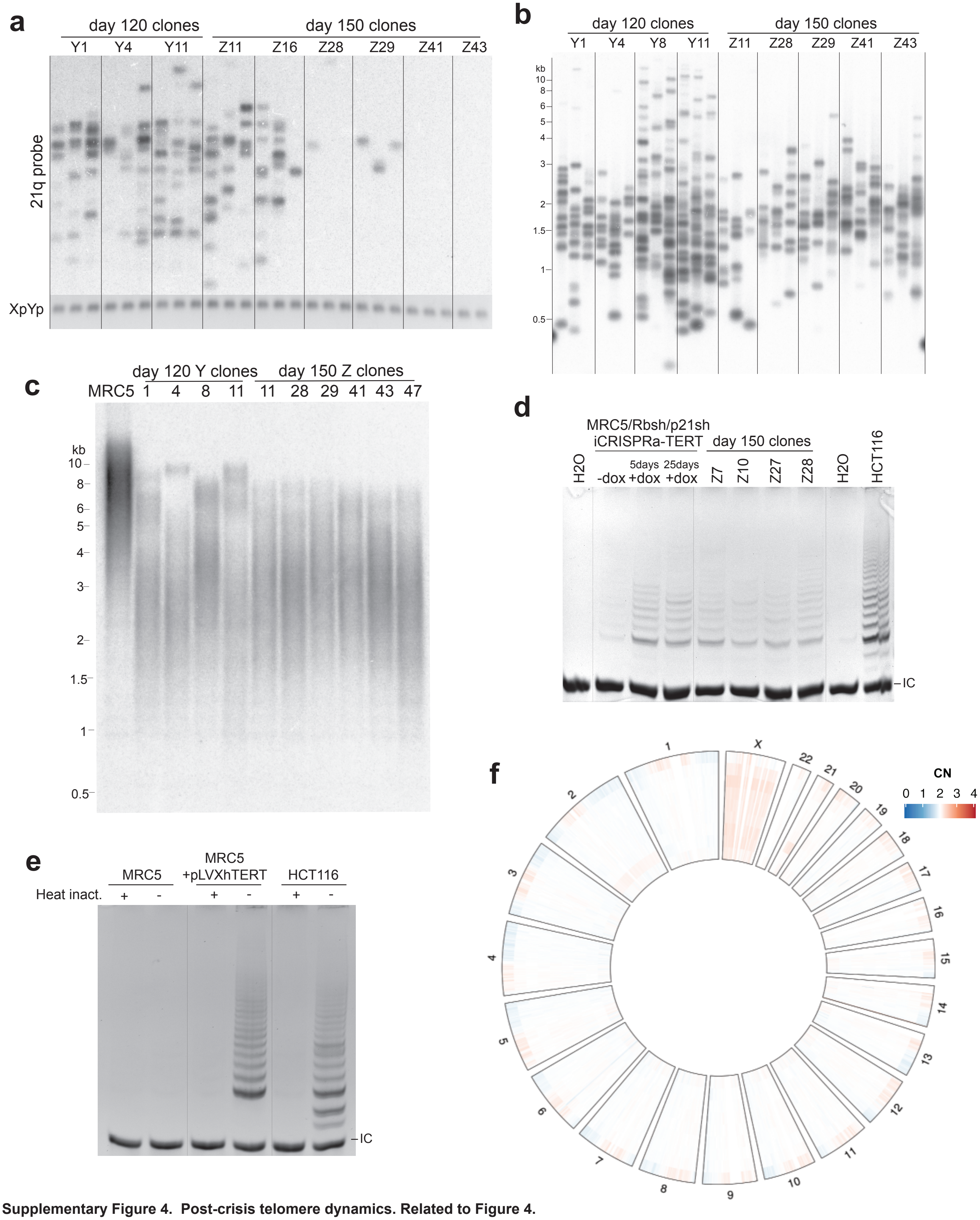

Supplementary Figure 4. Post-crisis telomere dynamics. Related to Figure 4.
a) Products of telomere fusion PCR on a panel of post-crisis clones (see Figure 4A). Telomere fusions are detected by hybridization to the 21q probe. The control XpYp band is detected with Ethidium bromide staining. The blot is representative of at least two experiments. **b)** STELA products from a panel of post-crisis clones from both time points. The blot is a representative of at least two experiments. **c)** Telomeric restriction fragment blot of DNA from MRC5 cells and a panel of post-crisis clones from both time points. The blot is a representative of at least two experiments. **d)** TRAP assay showing telomerase activity in MRC5/Rbsh/p21sh/iCRISPRa-TERT treated with doxycycline for the indicated number of days and a selection of post-crisis clones from the day 150 timepoint. HCT116 is included as a positive control. IC= internal control, 36 bp. The gel is a representative of at least two experiments. **e)** TRAP assay showing robust telomerase activity in MRC5 cells infected with retroviral pLVX-hTERT. IC= internal control, 36bp The gel is a representative of at least two experiments. **f)** Circular heatmap showing genome-wide binned purity- and ploidy-transformed read depth (in units of CN across 8 low pass WGS-profiled control CT clones). Heatmap rows correspond to concentric rings in the heatmap.

Dewhurst, Yao *et al*. Supplementary Figure 5

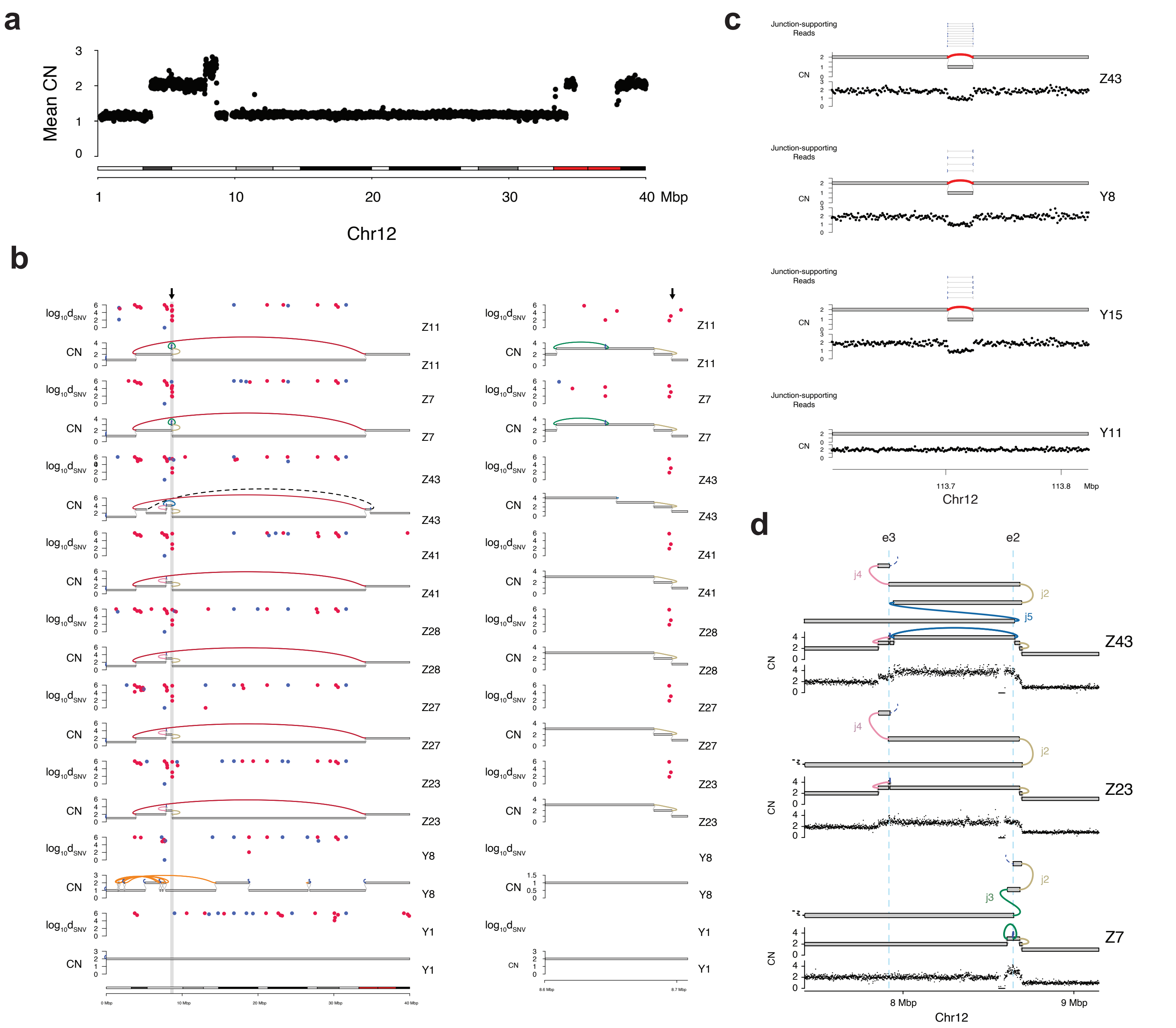

Supplementary Figure 5. WGS analysis showing genome alterations post crisis clones. Related to Figure 4 and 5.
a) Consensus copy number profile of 47 complex low pass WGS clones targeting chromosome 12p. **b)** Rainfall plot demonstrating SNV patterns as a function of GC vs AT reference nucleotide context, with y-axis showing the logarithm of the inter SNV distance. Highlighted region represents a GC strand coordinated cluster that is found across all four clones in the BFB cluster which harbour junction j2. **c)** Junction supporting read pairs and corresponding drop in coverage at the small deletion on chromosome 12q in representative BFB-like (Z43), chromothripsis-like (Y8), close-relative Y15, and lack of such evidence in distant Y11. **d)** Detailed JaBbA models and linear allele reconstruction at two distinct loose ends, e2 and e3.

Dewhurst, Yao *et al*. Supplementary Figure 6

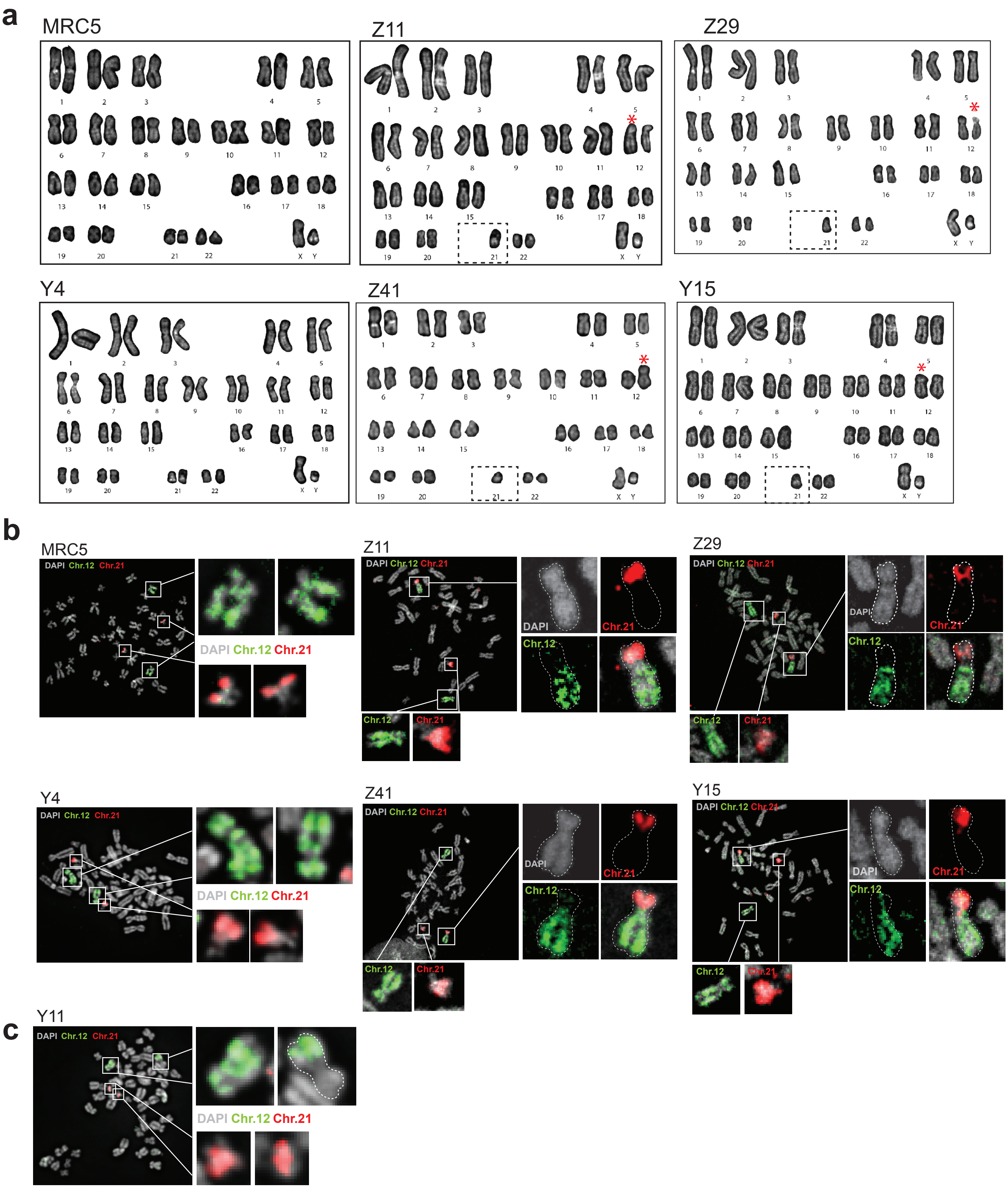

Supplementary Figure 6. Karyotype evolution in post-crisis clones. Related to Figure 6.
a) DAPI banded karyotypes from the MRC5 parental cell line and a selection of post-crisis clones. Marker chromosomes (derivative 12) are indicated with a red star. Dashed box indicates the absence of an intact copy of chromosome 21. **b)** Representative images of metaphase spreads from post-crisis clones hybridized with both 12 (green) or 21 (red) chromosome paints. DNA stained with DAPI (grey). **c)** Post-crisis clone Y11 hybridized with both 12 (green) or 21 (red) chromosome paints. DNA stained with DAPI (grey).

Dewhurst, Yao *et al*. Supplementary Figure 7

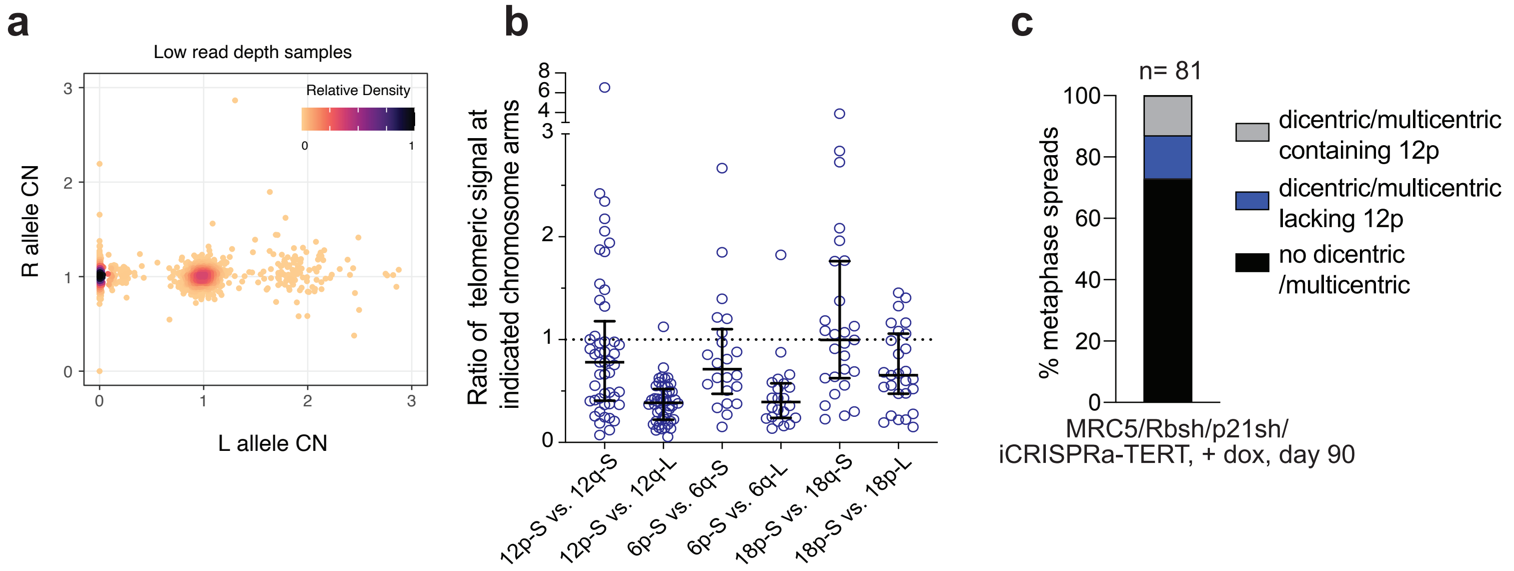

Supplementary Figure 7. Chromosome 12p allelic imbalance. Related to Figure 7.
a) Scatter plot showing purity- and ploidy-transformed L and R haplotype specific allelic read depth across 12p segments in low pass WGS-profiled post-crisis clones. **b)** Analysis showing that 12p, 6p and 18p arms contain the shortest telomere of those chromosomes. Comparison of the shortest (S) telomere end from each arm (p or q) to each other allele of that chromosome (i.e. q-short allele vs. p-short allele) across chromosome 12, 6 and 18 alleles. Ratio of telomeric intensity from TelG hybridization, as in Figure 7D (S= short allele, L= long allele, based on TelG intensity). Error bars represent median and interquartile ranges. Chromosome 12: n= 49 cells, chr.18: n=27, chr.6: n= 22. **c)** Fraction of dicentric or multicentric chromosomes containing 12p in MRC5/Rbsh/p21sh/iCRISPRa-TERT cells in crisis (with doxycycline at day 90) among all dicentric and multicentric chromosomes. Based on analysis of images as in Figure 7E. n=81 metaphases scored from two independent experiments.

Supplementary Table 1: SV40T immortalized pre-crisis and post-crisis telomerase positive cell lines

Name	Cell Line/Tissue Source	Telomerase status	Reference	Kind gift of
HA1 p15	Human Embryonic Kidney cells	Negative	(Counter et al., 1992)	Silvia Bacchetti/AdVec
SW13 PD73	IMR90 lung fibroblasts	Negative	(Shay and Wright, 1989)	Jerry Shay, UTSW
SW26 PD68.5	IMR90 lung fibroblasts	Negative	(Shay and Wright, 1989)	Jerry Shay, UTSW
SW39 PD72.5	IMR90 lung fibroblasts	Negative	(Shay and Wright, 1989)	Jerry Shay, UTSW
Bet3B p3	NHBE-10 bronchial epithelial cells	Negative	(Bryan et al., 1995)	Roger Reddel, CMRI
Bet3K p7	NHBE-10 bronchial epithelial cells	Negative	(Bryan et al., 1995)	Roger Reddel, CMRI
BFT3B p10	BF-10 bronchial fibroblasts	Negative	(Bryan et al., 1995)	Roger Reddel, CMRI
BFT3G p7	BF-10 bronchial fibroblasts	Negative	(Bryan et al., 1995)	Roger Reddel, CMRI
BFT3K p9	BF-10 bronchial fibroblasts	Negative	(Bryan et al., 1995)	Roger Reddel, CMRI
HA1-IM PD216	Human Embryonic Kidney cells	Positive (see ref.)	(Counter et al., 1992)	Silvia Bacchetti/AdVec
SW13 PD184	IMR90 lung fibroblasts	Positive (Fig.S1A)	(Shay and Wright, 1989)	Jerry Shay, UTSW
SW26 PD130+	IMR90 lung fibroblasts	Positive (Fig.S1A)	(Shay and Wright, 1989)	Jerry Shay, UTSW
SW39 PD130+	IMR90 lung fibroblasts	Positive (Fig.S1A)	(Shay and Wright, 1989)	Jerry Shay, UTSW
Bet3B p25 post-crisis	NHBE-10 bronchial epithelial cells	Positive (see ref.)	(Bryan et al., 1995)	Roger Reddel, CMRI
Bet3K p25 post-crisis	NHBE-10 bronchial epithelial cells	Positive (see ref.)	(Bryan et al., 1995)	Roger Reddel, CMRI
BFT3B p28 post-crisis	BF-10 bronchial fibroblasts	Positive (see ref.)	(Bryan et al., 1995)	Roger Reddel, CMRI
BFT3G p28 post-crisis	BF-10 bronchial fibroblasts	Positive (see ref.)	(Bryan et al., 1995)	Roger Reddel, CMRI
BFT3K p34 post-crisis	BF-10 bronchial fibroblasts	Positive (see ref.)	(Bryan et al., 1995)	Roger Reddel, CMRI

Supplementary Table 2: **Number of clones analyzed by high and low-pass WGS**

Name	Number Sequenced	
	Low Pass	High Pass
Parental cell lines	3	1
Day 120 (Y) clones	37	5
Day 150 (Z) clones	83	8
Control (CT) clones	8	0

Supplementary Table 3: Oligos used in this study

OLIGO NAME and SEQUENCE	SOURCE
TERT_gRNA_1- AGTCGCGGGGAAGTGTTGCA	This study
TERT_gRNA_2- ATCTGCCAGACAGAGTGCCG	This study
TERT_gRNA_3- TCGAATCGGCCTAGGCTGTG	This study
TERT_gRNA_4- GAAACTCGCGCCGCGAGGAG	This study
TTN_gRNA_1- CCTTGGTGAAGTCTCCTTTG	(Chavez et al., 2015)
TTN_gRNA_2- ATGTTAAAATCCGAAAATGC	(Chavez et al., 2015)
TTN_gRNA_3- GGGCACAGTCCTCAGGTTTG	(Chavez et al., 2015)
TTN_gRNA_4- ATGAGCTCTCTTCAACGTTA	(Chavez et al., 2015)
TERT qPCR forward- GGAGCAAGTTGCAAAGCATTG	This study
TERT qPCR reverse- TCCCACGACGTAGTCCATGTT	This study
TTN qPCR forward- TGTTGCCACTGGTGCTAAAG	This study
TTN qPCR reverse- ACAGCAGTCTTCTCCGCTTC	This study
β-actin qPCR forward-TGGATCAGCAAGCAGGAGTATG	This study
β-actin qPCR reverse- GCATTTGCGGTGGACGAT	This study
STELA telorette1- TGCTCCGTGCATCTGGCATCCCCTAAC	(Baird et al., 2003)
STELA telorette2- TGCTCCGTGCATCTGGCATCTAACCCT	(Baird et al., 2003)
STELA telorette3- TGCTCCGTGCATCTGGCATCCCTAACC	(Baird et al., 2003)
STELA telorette4-TGCTCCGTGCATCTGGCATCCTAACCC	(Baird et al., 2003)
STELA telorette5- TGCTCCGTGCATCTGGCATCAACCCTA	(Baird et al., 2003)
STELA telorette6- TGCTCCGTGCATCTGGCATCACCCTAA	(Baird et al., 2003)
STELA XpYpE2- TTGTCTCAGGGTCCTAGTG	(Baird et al., 2003)
STELA teltail - TGCTCCGTGCATCTGGCATC	(Baird et al., 2003)
STELA XpYpB2- TCTGAAAGTGGACCTATCAG	(Baird et al., 2003)
Fusion PCR XpYpM- ACCAGGTTTTCCAGTGTGTT	(Capper et al., 2007)
Fusion PCR 17p6- GGCTGAACTATAGCCTCTGC	(Capper et al., 2007)
Fusion PCR 21q4- GGGACATATTTTGGGGTTGC	(Capper et al., 2007)
Fusion PCR XpYpc2tr- GCTATGGCTTCTTGGGGC	(Capper et al., 2007)
Fusion PCR 21q-seq-rev2-ACACAGAAGGTTGATATACACAG	(Capper et al., 2007)
