# Supplementary material for: Structural variant evolution after telomere crisis
Source: Nat Commun. 2021 Apr 7;12:2093. doi: 10.1038/s41467-021-21933-7 (PMC8027843; doi:10.1038/s41467-021-21933-7)
Supplement: Supplementary file 2 — Reporting Summary [file 41467_2021_21933_MOESM2_ESM.pdf]

nature research

Corresponding author(s): Marcin Imielinski

Last updated by author(s): Jan 14, 2021

Reporting Summary

Nature Research wishes to improve the reproducibility of the work that we publish. This form provides structure for consistency and transparency in reporting. For further information on Nature Research policies, see our Editorial Policies and the Editorial Policy Checklist.

Statistics

For all statistical analyses, confirm that the following items are present in the figure legend, table legend, main text, or Methods section.

n/a	Confirmed	
☐	☒	The exact sample size (n) for each experimental group/condition, given as a discrete number and unit of measurement
☐	☒	A statement on whether measurements were taken from distinct samples or whether the same sample was measured repeatedly
☐	☒	The statistical test(s) used AND whether they are one- or two-sided<br>*Only common tests should be described solely by name; describe more complex techniques in the Methods section.*
☒	☐	A description of all covariates tested
☒	☐	A description of any assumptions or corrections, such as tests of normality and adjustment for multiple comparisons
☐	☒	A full description of the statistical parameters including central tendency (e.g. means) or other basic estimates (e.g. regression coefficient) AND variation (e.g. standard deviation) or associated estimates of uncertainty (e.g. confidence intervals)
☐	☒	For null hypothesis testing, the test statistic (e.g. F, t, r) with confidence intervals, effect sizes, degrees of freedom and P value noted<br>*Give P values as exact values whenever suitable.*
☒	☐	For Bayesian analysis, information on the choice of priors and Markov chain Monte Carlo settings
☐	☒	For hierarchical and complex designs, identification of the appropriate level for tests and full reporting of outcomes
☐	☒	Estimates of effect sizes (e.g. Cohen's d, Pearson's r), indicating how they were calculated

Our web collection on statistics for biologists contains articles on many of the points above.

Software and code

Policy information about availability of computer code

Data collection	Microscopy images were acquired using SoftWoRx (v.7.0.0) on a DeltaVision microscope (Applied Precision) equipped with a cooled charge-coupled device camera (DV Elite CMOS Camera), with a PlanApo 60× 1.42 NA objective (Olympus America). Real-time qPCR data was collected on a Life Technologies QuantStudio 12K machine. Southern blots were scanned on a STORM alpha imager.
Data analysis	Data was analyzed with the following software: FIJI (v.2.0.0-rc-69/1.52p), PRISM (v. 8.4.0), bwa (version 0.7.10-r789, http://bio-bwa.sourceforge.net/), R (v3.6.1, http://www.r-project.org/), Bioconductor (version 3.10, https://www.bioconductor.org/), GATK (version 3.7-0-gcfedb67, https://software.broadinstitute.org/gatk/), Picard (version 3.7-0-gcfedb67, https://broadinstitute.github.io/picard/), Strelka2 (https://github.com/Illumina/strelka), SvAbA (version 1.1.3, https://github.com/walaj/svaba), GRIDSS (version 2.9.4, https://github.com/PapenfussLab/gridss), samblaster (version 0.1.22, https://github.com/GregoryFaust/samblaster), samtools (version 1.9, http://www.htslib.org/), custom codes JaBbA build 7926cc7, gGnome build 7f5bf56, dryclean build 6d2bced, fragCounter build 575af99, rSeqLib build 23fbaf0, skitools build 61187fa, gUtils build 449ab2a, gTrack build 947c35c; all available at https://github.com/mskilab. Analysis code to generate the figures in the paper is available on request.

For manuscripts utilizing custom algorithms or software that are central to the research but not yet described in published literature, software must be made available to editors and reviewers. We strongly encourage code deposition in a community repository (e.g. GitHub). See the Nature Research guidelines for submitting code & software for further information.

Data

Policy information about availability of data

All manuscripts must include a data availability statement. This statement should provide the following information, where applicable:

- Accession codes, unique identifiers, or web links for publicly available datasets
- A list of figures that have associated raw data
- A description of any restrictions on data availability

Whole genome sequencing data has been deposited to the sequence read archive as aligned .bam files (https://www.ncbi.nlm.nih.gov/sra) under the BioProject accession PRJNA693405 (https://www.ncbi.nlm.nih.gov/sra/?term=PRJNA693405).

Field-specific reporting

Please select the one below that is the best fit for your research. If you are not sure, read the appropriate sections before making your selection.

☒ Life sciences ☐ Behavioural & social sciences ☐ Ecological, evolutionary & environmental sciences

For a reference copy of the document with all sections, see nature.com/documents/nr-reporting-summary-flat.pdf

Life sciences study design

All studies must disclose on these points even when the disclosure is negative.

Sample size	No formal statistical methods were used to determine sample size. For in vitro assays, sufficient numbers of technical and biological replicates were performed to reduce biological and technical variability, according to prior experience and are standard in the field (see e.g. Mirman et al., Nature, 2018). For sequencing of post-crisis clones, sample size was limited by the number of viable post-crisis clones that could be isolated, and by the expense of sequencing.
Data exclusions	No data were excluded.
Replication	The entire long-term culture experiment was performed three times from start to finish in order to derive true biological replicates. Experiments with less than three replicates were limited by the amount of biological material available in each repeat of the experiment. At least two biological replicates were performed for each in vitro experiment at each time point with consistent results, and in each case the exact n number is reported in the relevant figure legends.
Randomization	We report on in vitro experiments where randomization is generally not required. At the start of the long-term culture, cells were randomly allocated to be treated or not treated with doxycycline. Each long-term replicate was randomly split into dox treated or not. After the start of the experiment, all samples were necessarily either dox-treated or not and further randomization was not possible. Samples were grouped according to time points after the beginning of the experiment.
Blinding	There was no blinding of any group allocation during data collection. It was deemed unnecessary since all data collected was quantitative (for example scored by algorithm). Furthermore, untreated cells were progressing into telomere crisis, and were therefore morphologically distinct from treated cells, such that blinding would have been impossible.

Reporting for specific materials, systems and methods

We require information from authors about some types of materials, experimental systems and methods used in many studies. Here, indicate whether each material, system or method listed is relevant to your study. If you are not sure if a list item applies to your research, read the appropriate section before selecting a response.

Materials & experimental systems

n/a	Involved in the study	
☐	☒	Antibodies
☐	☒	Eukaryotic cell lines
☒	☐	Palaeontology and archaeology
☒	☐	Animals and other organisms
☒	☐	Human research participants
☒	☐	Clinical data
☒	☐	Dual use research of concern

Methods

n/a	Involved in the study	
☒	☐	ChIP-seq
☒	☐	Flow cytometry
☒	☐	MRI-based neuroimaging

Antibodies

Antibodies used	Mouse monoclonal anti-Cas9 (7A9-3A3), Cell Signaling Technology Cat#14697S 1:1000<br>Mouse monoclonal anti-γ-tubulin Sigma Cat#T5326 1:1000

	Mouse monoclonal anti- Human Retinoblastoma protein BD Pharmigen Cat#554136 1:500<br>Mouse monoclonal anti-p21 (F-5) Santa Cruz Cat#sc-6246 1:200<br>Rabbit monoclonal anti-53BP1 Abcam Cat#ab-175933 1:1000<br>F(ab')2-Goat anti-Rabbit IgG (H+L) Cross-Adsorbed Secondary Antibody Alexa Fluor 488 ThermoFisher Cat#A-11070 1:500<br>Sheep polyclonal anti-dioxigenin-Rhodamine Roche Cat# 11207750910 1:400<br>Chicken Avadin-FITC VWR Cat# CAP21221 1:400<br>Amersham ECL Mouse IgG, HRP-linked whole Ab (from sheep) NXA931<br>Amersham ECL Rabbit IgG, HRP-linked whole Ab (from donkey) NA934
Validation	Cas9 Ab: validated as only detects exogenous bacterial protein, see Figure 2A - no signal detected in uninfected cells, also see data on the manufacturers website and in papers such as PMID: 32380416.<br>Rb and p21 specificity validated by shRNA mediated knock-down experiments, see Supp Fig 2A. Rb antibody: see information on manufacturers website: https://www.bdbiosciences.com/ds/pm/tds/554136.pdf and publications such as http://genesdev.cshlp.org/content/7/9/1654.long. For p21 see the manufacturers website https://www.scbt.com/p/p21-antibody-f-5.<br>53BP1 Ab has been validated previously in our lab using knock-out or knock-down cell lines, see e.g. Mirman et al., Nature 2018.<br>No additional validation was carried out for tubulin loading control, information on manufacturers website, and publications such as https://www.sigmaaldrich.com/catalog/papers/22806905.

Eukaryotic cell lines

Policy information about cell lines

Cell line source(s)	MRC5 human lung fibroblasts (CCL-171), Phoenix-ampho (CRL-3213), RPE-1 hTERT (CRL-4000), HCT-116 (CCL-247) and U2-OS (HTB-96) cells were obtained from ATCC for this study. 293-FT cells were obtained from ThermoFisher. HA-1M cells were a kind gift of Silvia Bacchetti, SW13/26/39 cells were a kind gift of Jerry Shay, and Bet-3B/3K and BFT-3B/G/K cells were a kind gift of Roger Reddel. Only SW13/26/39 were cultured in our lab - DNA was extracted directly from the other cell line vials.
Authentication	None of the cell lines used were independently authenticated for this specific study. MRC5 cells were obtained freshly from ATCC for this study.
Mycoplasma contamination	Cell lines were not specifically tested for mycoplasma during this study, but immuno-fluoresence experiments which can detect mycoplasma incidently were routinely negative.
Commonly misidentified lines<br>(See ICLAC register)	No commonly misidentified cell lines were used in this study.
